# IN THE WEEDS: UNDERSTANDING CANNABIS USE AMONG OLDER ADULTS

**DOI:** 10.1093/geroni/igac059.2152

**Published:** 2022-12-20

**Authors:** Isabelle Sheck, H Shellae Versey

**Affiliations:** Fordham University, Bronx, New York, United States; Fordham University, Bronx, New York, United States

## Abstract

The existing literature from the past decade shows an increase in the prevalence of cannabis use among older adults (OA) aged 50-65. Most commonly, studies have reported OA using cannabis to manage symptoms related to chronic pain, arthritis, insomnia or disordered sleep, and anxiety. Recent recreational cannabis legislation in several states has also contributed to increasing rates of cannabis use among older adults. Despite an increase in recreational and medicinal cannabis consumption, few studies have focused on cannabis attitudes and behaviors among older adults. Within the past ten years, studies reporting cannabis behavior have been limited to samples of younger adults. This paper provides a comprehensive review of qualitative and quantitative studies of cannabis use among older adults (OA). Additional research detailing the characteristics of cannabis use among OA is needed, including frequency, motivation, type of cannabis (medicinal/recreational), route of administration, suppliers, and social networks associated with cannabis use. This systematic review adds to our knowledge about aging and substance use addressing a gap in the literature regarding drivers of cannabis use among OA. Implications of this research extend to gerontological, public health, and community research as cannabis continues to become more easily accessible in various states.

